# Preparation and Gas Separation of Amorphous Silicon Oxycarbide Membrane Supported on Silicon Nitride Membrane

**DOI:** 10.3390/membranes14030063

**Published:** 2024-03-02

**Authors:** Hengguo Jin, Xin Xu

**Affiliations:** CAS Key Laboratory of Materials for Energy Conversion, Department of Materials Science and Engineering, University of Science and Technology of China, Hefei 230026, China; jhg@mail.ustc.edu.cn

**Keywords:** silicon oxycarbide, silicon nitride, pre-ceramic polymers, spin coating, pyrolysis, gas separation

## Abstract

An amorphous silicon oxycarbide membrane supported on a silicon nitride membrane substrate was prepared. A starting suspension containing polyhydromethylsiloxane (PHMS), tetramethyltetravinyl-cyclotetrasiloxane (TMTVS) and a platinum catalyst was first prepared and spin-coated on a silicon nitride membrane, and then the suspension was cross-linked and cured, followed by pyrolyzing at 1000 °C under a flowing Ar atmosphere. A dense amorphous silicon oxycarbon ceramic membrane with a thickness of about 1.8 µm was strongly bonded to the Si_3_N_4_ membrane substrate. The single gas permeation of H_2_ and CO_2_ indicated that the ideal permeation selectivity of H_2_/CO_2_ was up to 20 at 25 °C and 0.5 MPa with good long-term stability, indicating the potential application of the obtained membrane for hydrogen purification.

## 1. Introduction

With an ever-evolving society, the world is facing energy and climate issues [1], which urgently need to be resolved. As a renewable, non-toxic gas [2], hydrogen has a high calorific value [3] and does not produce greenhouse gases [4], so it has a wide range of applications in energy and climate issues [5]. The production of hydrogen gas has gained great attention [6,7,8,9,10,11], while the purification of hydrogen is also a problem worthy of attention [12,13,14].

Hydrogen purification methods are mainly pressure swing adsorption [15,16,17,18], cryogenic distillation [13,19] and membrane separation [20]. Compared with the other two methods, the membrane separation method has received more and more attention due to its simple operation [21], low energy consumption [22,23,24], low investment cost [25] and high efficiency [26,27]. Many membranes have been studied [28], such as graphene oxide (GO) [29,30], polymers [31,32,33,34,35], pure metal membranes [28], mixed-matrix membranes [28] and zeolitic imidazolate frameworks (ZIFs) [36,37,38,39,40]. The porous structure of carbon membranes partially collapses at high temperatures [28]. Polymer membranes have low mechanical strength, are not resistant to corrosion and are highly sensitive to compaction [13]. The appearance of hydrogen embrittlement will cause cracks on the surface of metal membranes [28]. In mixed-matrix membranes, most fillers are expensive, and further study on the scaling up of the processes from the lab scale to the pilot scale is needed [28]. And the thickness of defect-free ZIF membranes used for separation is often very thick [36]. On the contrary, inorganic membranes are gaining more and more attention due to their excellent mechanical strength [41], strong corrosion [42] and high temperature resistance. Polymer-derived ceramics, such as SiOC, SiCN and SiOCN, have attracted great interest due to their low-temperature ceramization, molecular design, good manufacturability and processability [43,44,45].

In polymer-derived SiOC membranes, the SiOC network is generated by the substitution of two divalent oxygen ions by one tetravalent carbon ion within the SiO_2_ network. CSi_4_ units are locally formed to strengthen the network. They are most widely studied due to their low cost, high ceramic yield, moderate pyrolysis temperature and ease of control over curing and molding [46,47].

Many studies on SiOC have been carried out, such as the use of polymer-derived SiOC ceramic membranes for oil–water separation and membrane distillation [48], and polymer-derived SiOC microbeads have been used to make anodes for high-performance lithium-ion batteries [49]. SiOC was formed by pyrolysis using polyhydromethylsiloxane (PHMS) and tetramethyltetravinylcycletetrasiloxane (TMTVS) as the precursors of Si, which was amorphous, where oxygen and carbon were simultaneously connected to silicon. Generally, the application was mainly carried out by loading the precursor membrane layer on a traditional ceramic membrane support [50]. Prasad et al. [13] used vinyl-functionalized polysiloxane XP RV 200 as the precursor and successfully prepared an amorphous SiOC membrane on the substrate of an alumina tube by a dip coating method.

In this study, we successfully prepared an amorphous silicon oxycarbide membrane supported on a silicon nitride ceramic membrane substrate because silicon nitride has similar chemical bonds with SiOC, which was beneficial for uniform growth and the strong adhesion of the obtained SiOC membrane. The Si_3_N_4_ membrane contained large, straight finger-like voids, which favored the flow of gas. The prepared membrane showed good performance in the selective permeability of hydrogen and carbon dioxide.

## 2. Materials and Methods

Polyhydromethylsiloxane, 1,3,5,7-tetramethyl-1,3,5,7-tetravinylcyclo-tetrasiloxane and platinum divinyl-tetramethyl-disiloxane complex (Pt-C) were all purchased from Shanghai Guiyou New Material Technology Ltd., Shanghai, China. High-purity argon and nitrogen (greater than or equal to 99.999%) were from Nanjing Shangyuan Industrial Gas Factory.

A silicon nitride ceramic membrane with a diameter of 2.5 cm and a thickness of 1 mm was prepared by phase-inversion tape casting and pressureless sintering [51]. The Si_3_N_4_ membrane was polished with sandpaper, followed by washing with a hot sodium hydroxide solution [52]. The cleaned silicon nitride membrane was firmly fixed on a suction cup.

**Figure 1:**
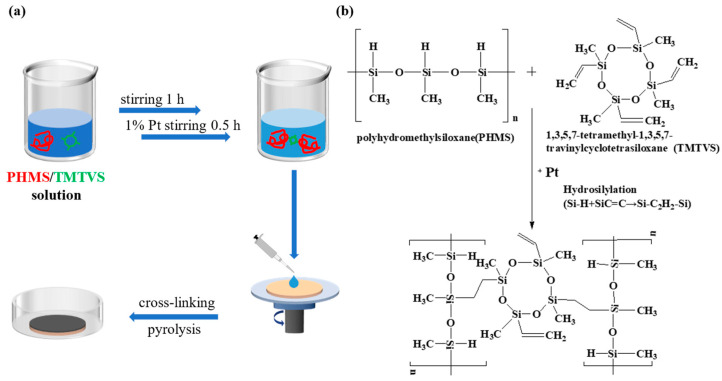
(**a**) Preparation diagram of composite ceramic membrane. PHMS and TMTVS correspond to the red and green lines, respectively. (**b**) Schematic diagram of the hydrosilanization reaction between PHMS and TMTVS.

The preparation process for the ceramic separation membrane is shown in Figure 1a. PHMS (the precursor polymer) and TMTVS (the crosslinking agent) with a mass ratio of 1:1 were first mixed in a magnetic stirrer for one hour. Then, the Pt-C catalyst (1wt% of PHMS and TMTVS) was added and stirred for half an hour to make the hydrosilylation of Si-H and CH_2_=CH_2_ groups from PHMS and TMTVS more thorough, as shown in Figure 1b. The starting suspension was rotationally sprayed onto a silicon nitride membrane using an EZ4 spin coat (EZ4-S, Lebo Science, Wuxi, China), which rotated at 400 r/min and dropped 2 mL each time, followed by curing at a constant temperature in a humidity box (HSB-80L, Hefei Anke Environmental Testing Equipment Co., Ltd., Hefei, China). Subsequently, the ceramic membrane was heated in an oven at 80 °C for 12 h and then at 120 °C for another 12 h, and the polymeric gel was cross-linked. Afterward, the resulting PSO gel (cross-linked curing from PHMS, TMTVS and Pt-C) was pyrolyzed at different temperatures between 500 and 1000 °C in a flowing argon atmosphere for 3 h with a heating rate of 5 °C/min. The above procedure was repeated once to obtain the composite membrane. 

The crystalline phases were identified by X-ray diffraction (XRD, Philips, PW 1700, Eindhoven, Netherlands). The cross-section and surface morphology of the final membrane were measured with a scanning electron microscope (SEM, JEOL JSM—6390LA, Tokyo, Japan), equipped with an EDS device for elemental analysis. The pyrolysis behavior was also examined through a differential scanning calorimetry–thermogravimetric analysis (DSC-TGA, SDT Q600, TA Instruments, New Castle, DE, USA) under a nitrogen atmosphere. The binding energies were characterized by X-ray photoelectron spectroscopy (XPS, Kratos Axis supra, Shimadzu, Kyoto, Japan). The specific surface areas and pore-size distributions of the ceramics were determined by a N_2_ adsorption–desorption technique using an ASAP 2460 V3.00H instrument. Fourier transform infrared spectroscopy (FTIR, Nicolet6700, Thermo Fisher, Waltham, MA, USA) was used to characterize the chemical bonds in polymer gels.

**Figure 2:**
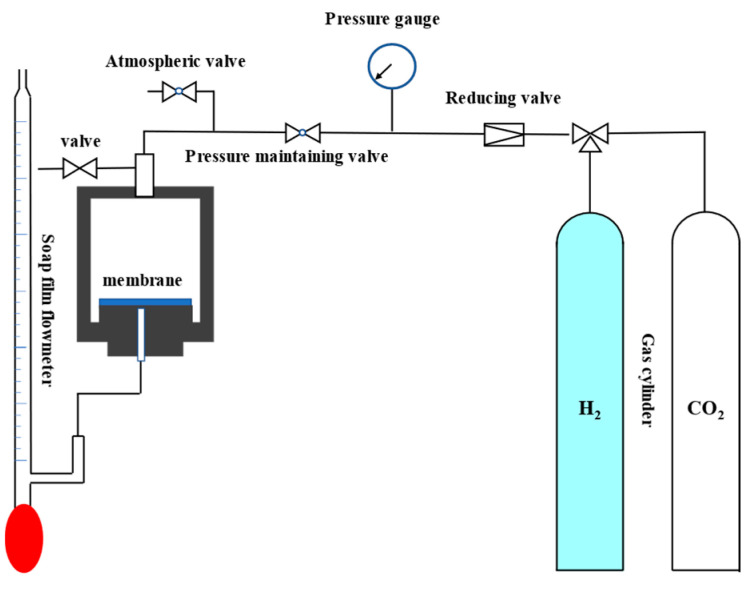
Gas permeation test equipment for the prepared ceramic membrane.

The separation device and test method for a single gas were reported previously [36]. The test instrument is shown in Figure 2. The pressure difference between the feed side and the permeate side of the membrane was 0.5 MPa. The single H_2_ (0.29 nm) and CO_2_ (0.33 nm) gas permeability was measured as a function of time at 25 °C, and the ideal gas separation factor of H_2_ and CO_2_ was the ratio of the permeability of the two gases.

## 3. Results

### 3.1. Characterization of SiOC Ceramics

**Figure 3:**
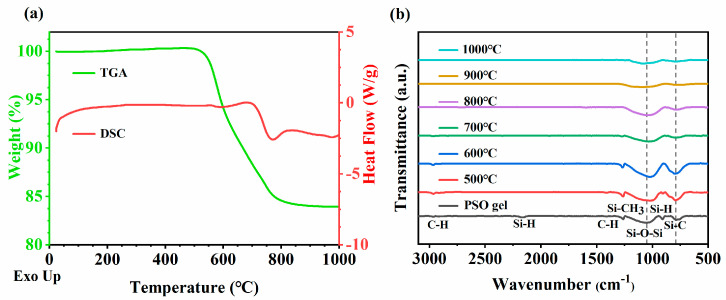
(**a**) The DSC-TG analysis of PSO gel. (**b**) Fourier transform infrared spectroscopy (FTIR) of PSO gel before and after pyrolysis at different temperatures.

The DSC-TG analysis of the PSO gel was carried out in a nitrogen atmosphere, as shown in Figure 3a. The gel started to decompose at around 500 °C, suggesting a high level of stability in the gelling process [49]. The pyrolysis curve showed that a strong weight loss occurred in the temperature range of 500 to 800 °C, which might be due to the release of low-molecular-weight oligomers, such as hydrogen, methane, etc. The strong exothermic peak close to 780 °C indicates the transition from the PSO gel to SiOC ceramics. The total weight loss indicated that the ceramic yield was about 83% at 1000 °C [49].

Figure 3b shows the FTIR spectra after pyrolysis at different temperatures. There are always characteristic absorption peaks at approximately 790 cm^−1^ and 1050 cm^−1^, which were attributed to the stretching vibration of the Si-C bond and Si-O-Si bond, respectively. It is worth mentioning that Si-O-Si characteristic peaks always move in the direction of low wave number after pyrolysis. The PSO gel has peaks at 2960 cm^−1^, 2160 cm^−1^, 1410 cm^−1^, 1260 cm^−1^ and 910 cm^−1^, corresponding to the characteristic absorption peaks of C-H, Si-H, C-H, Si-CH_3_ and Si-H bonds, respectively. No absorption peak of the C=C bond was seen near 1600 cm^−1^ because the C=C bond in TMTVS reacted with the Si-H bond in PHMS to form a Si-CH_2_-CH_2_-Si bond, which was completely consumed. Si-H disappeared at temperatures lower than 600 °C through the following equation [31]:

Si-CH_3_ + Si-H → Si-CH_2_-Si + H_2_ (1)

As the temperature increased, the peak of Si-CH_3_ gradually decreased and disappeared at 800 °C through Equation (2):

Si-CH_3_ + Si-CH_3_ → Si-CH_2_-Si + CH_4_ (2)

A further condensation reaction of methylene was expected to occur at high temperatures, leading to the formation of a three-dimensional network structure and the release of CH_4_. 

**Figure 4:**
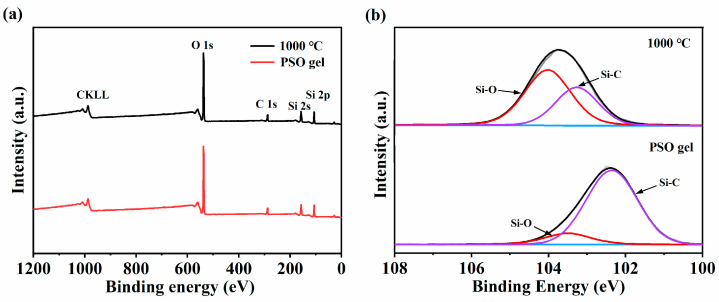
(**a**) XPS spectrum of PSO gel and SiOC membrane pyrolyzed at 1000 °C. (**b**) XPS fitting curve of Si2p.

Figure 4a presents the XPS analysis of the PSO gel and the SiOC membrane pyrolyzed at 1000 °C, where the photoelectron energy absorption peaks of Si, O and C could be detected. As shown in Figure 4b, the Si2p peak of the SiOC membrane can be decomposed into two peaks, namely 104.0 eV and 103.3 eV, attributed to the Si-O and Si-C bonds, respectively, which is in good agreement with the FTIR absorption spectrum (Figure 3b). Compared with the PSO gel, the SiOC ceramics exhibited a significantly decreased intensity of Si-C bonds, which could be attributed to the dissociation of Si-C [53].

**Figure 5:**
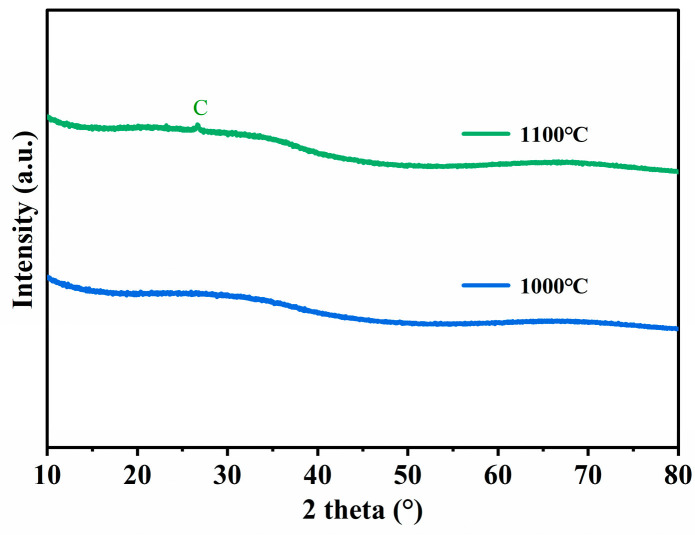
XRD diagrams of SiOC membrane pyrolyzed at 1000 °C and 1100 °C.

Figure 5 shows the XRD diagram of the SiOC membrane pyrolyzed at 1000 °C and 1100 °C. When the treatment temperature was increased to 1100 °C, small graphite peaks appeared, indicating the decomposition of SiOC, while all the membranes obtained at temperatures lower than 1000 °C were amorphous [54]. 

**Figure 6:**
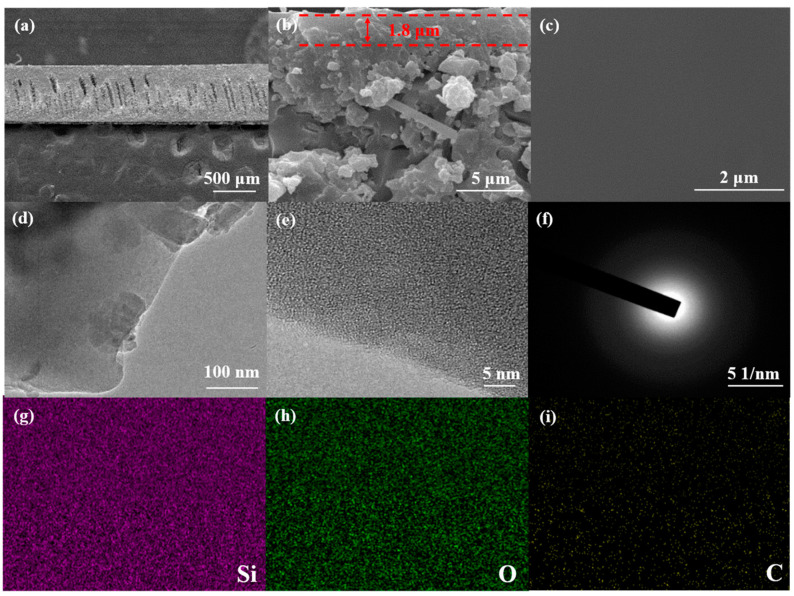
(**a**) Cross-section SEM of the SiOC membrane on the Si_3_N_4_ ceramic membrane; (**b**) fracture and (**c**) upper surface of the SiOC membrane; (**d**) TEM and (**e**) HRTEM of the SiOC membrane; (**f**) SAED of the SiOC membrane; and (**g**–**i**) EDS mapping of (**c**).

Figure 6a presents the SEM image of the cross section of the SiOC membrane on the Si_3_N_4_ ceramic membrane. The flat silicon nitride ceramic membrane revealed the existence of finger holes, which could significantly reduce the gas flow resistance. Figure 6a also shows the presence of pore penetration in the composite membrane, which can enhance the adhesion between the separation layer and the support layer through mechanical interlocking [55]. Figure 6b,c show the fracture and upper surface of the SiOC membrane. It can be clearly seen that a uniform and smooth SiOC membrane with a thickness of approximately 1.8 µm was successfully deposited on the surface of the Si_3_N_4_ membrane, which could be further confirmed by TEM and HRTEM (Figure 6d–e). In addition, it can also be seen from Figure 6b that the separation layer of the amorphous silicon oxycarbide membrane supported by silicon nitride is dense, defect-free and has good adhesion to the support body, which will be demonstrated by long-term permeation measurements of H_2_ and CO_2_ [55]. The SAED image in Figure 6f also indicates that the SiOC membrane was amorphous, retaining the memory of the PSO gel [56]. Figure 6g–i show an EDS map of Figure 6c, indicating the uniform distribution of Si, O and C. The morphology of the membrane indicates that multiple spin-coating pyrolysis ensures the compactness of the SiOC membrane by eliminating the small pores during gel formation.

**Figure 7:**
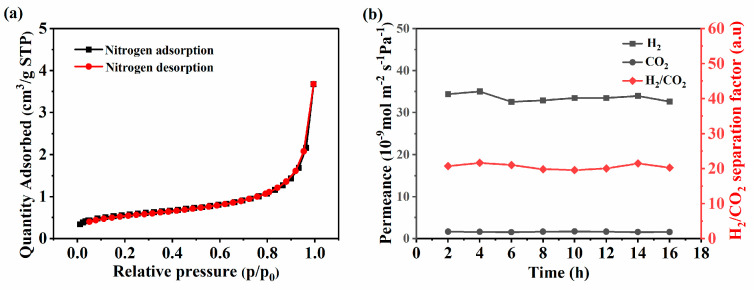
(**a**) Nitrogen adsorption and desorption isotherm of the SiOC membrane. (**b**) The gas permeability of H_2_/CO_2_ as a function of the penetration time.

Figure 7a shows the nitrogen adsorption isotherm at −196 °C of the SiOC membrane obtained at 1000 °C. The low nitrogen adsorption capacity in the relative pressure range of 0.01 to 1.0 indicates that the SiOC membrane was non-porous, which may be caused by the compactness of the SiOC structure [31,53]. So, the permeance of gas through the membrane should obey a mechanism involving jumps between solubility sites for non-porous membranes [57].

### 3.2. Gas Separation Performance of Composite Membrane

Figure 7b shows the gas permeability as a function of the penetration time. The single gas permeability of H_2_ (kinetic diameter 0.289 nm) and CO_2_ (kinetic diameter 0.330 nm) was measured at 25 °C and 0.5 MPa. H_2_ showed a stable H_2_ permeation of 3.26 × 10^−8^ mol m^−2^ Pa^−1^ s^−1^, which is much higher than 1.61 × 10^−9^ mol m^−2^ Pa^−1^ s^−1^ for CO_2_. This also indicated that the synthesized membrane was very stable. The permeability of the gas depended mainly on the kinetic diameter of the gas rather than the molecular weight [14,21]. The ideal separation factor for H_2_ and CO_2_ was about 20, which was much higher than the corresponding ideal Knudsen diffusion selectivity (4.69), indicating that the membrane had molecular sieving behavior. The prepared SiOC membrane was compared in detail with the reported membranes, as shown in Table 1. It can be clearly seen that the SiOC membrane showed good selectivity. The good performance of the composite ceramic membrane could be attributed to the good compatibility between SiOC and silicon nitride and the formation of strong chemical bonds at the interface. Future research will focus on regulating the structure to adapt to different gases and their separation ability at high temperatures.

## 4. Conclusions

We used the spin-coating method to load the amorphous SiOC membrane on the Si_3_N_4_ membrane without using mesoporous materials as the transition layer. The prepared composite membrane showed good molecular sieving ability, which benefited from the dense SiOC ceramic membrane. The gas separation factor of H_2_ and CO_2_ was about 20, which is much higher than the Knudsen diffusion, 4.69. Therefore, SiOC ceramic membranes have a certain practicability for waste gas treatment and gas separation.

## Figures and Tables

**Table 1:** The ideal selectivity of the SiOC membrane in this work and various membranes previously reported.

Membrane	Base	Thickness (μm)	Temperature (°C)	H_2_ Permeability (mol m^−2^ Pa^−1^s^−1^)	Selectivity (H_2_/CO_2_)	Ref.
SiOC	Si_3_N_4_	~1.8	25	3.26 × 10^−8^	~20	This work
SiOC	Al_2_O_3_	~0.3	300	1.78 × 10^−8^	~10	[13]
BTESE	Al_2_O_3_	N/A	200	3.75 × 10^−7^	7.3	[19]
MoS_2_	Al_2_O_3_	0.06	35	8.21 × 10^−7^	4.4	[21]
Zeolite	Al_2_O_3_	20~30	35	2.0 × 10^−10^	24	[22]
SiOC	Al_2_O_3_ SiO_2_ ZrO_2_	N/A	200	8.90 × 10^−7^	<10	[31]
ZIF-8	Si_3_N_4_	>60	25	8.35 × 10^−7^	~7.3	[36]

N/A relevant data are not available.

## Data Availability

Data are contained within the article.
